# Cyanobacterial Hydrogenases and Hydrogen Metabolism Revisited: Recent Progress and Future Prospects

**DOI:** 10.3390/ijms160510537

**Published:** 2015-05-08

**Authors:** Namita Khanna, Peter Lindblad

**Affiliations:** Microbial Chemistry, Department of Chemistry—Ångström Laboratory, Uppsala University, Box 523, Uppsala SE-75120, Sweden; E-Mail: namitakhanna1@gmail.com

**Keywords:** biohydrogen, bidirectional Hox hydrogenase, cyanobacteria, ferredoxin, [FeFe] hydrogenase, [NiFe] hydrogenase

## Abstract

Cyanobacteria have garnered interest as potential cell factories for hydrogen production. In conjunction with photosynthesis, these organisms can utilize inexpensive inorganic substrates and solar energy for simultaneous biosynthesis and hydrogen evolution. However, the hydrogen yield associated with these organisms remains far too low to compete with the existing chemical processes. Our limited understanding of the cellular hydrogen production pathway is a primary setback in the potential scale-up of this process. In this regard, the present review discusses the recent insight around ferredoxin/flavodoxin as the likely electron donor to the bidirectional Hox hydrogenase instead of the generally accepted NAD(P)H. This may have far reaching implications in powering solar driven hydrogen production. However, it is evident that a successful hydrogen-producing candidate would likely integrate enzymatic traits from different species. Engineering the [NiFe] hydrogenases for optimal catalytic efficiency or expression of a high turnover [FeFe] hydrogenase in these photo-autotrophs may facilitate the development of strains to reach target levels of biohydrogen production in cyanobacteria. The fundamental advancements achieved in these fields are also summarized in this review.

## 1. Introduction

A complete de-carbonization of the transport and electricity generation sector is required to avoid drastic climatic changes. Hydrogen appears to be a promising harbinger of this clean energy revolution. Due to recent major technical advancements, renewables are no longer considered as an expensive, immature technological alternative to limit carbon emissions; they are becoming increasingly cost competitive in several countries around the globe. Automakers have already launched hydrogen-powered vehicles that emit water as the only by product [1]. The traditional means to manufacture hydrogen employs natural gas, electricity, and conventional carbon emitting power plants. However, in nature, microorganisms possess an inherent capacity to turnover hydrogen via complex, novel enzymes known as hydrogenases. Hydrogenases are integrated into the cellular network such that they can dispose of excess reductants to generate a cellular waste product “hydrogen” [2]. Among the various hydrogen-producing microbes, photosynthetic cyanobacteria share similarity to the organisms of the eukaryotic world in possessing two photosystems [3,4]. The presence of photosystem II (PSII) empowers oxygen evolution by splitting of water molecules using light energy. Photosynthetic organisms possessing hydrogen related enzymes such as the bidirectional Hox hydrogenase or nitrogenase can directly employ reductant generated from the splitting of water to produce hydrogen [5,6]. In this respect, cyanobacteria are the optimal candidates because they can directly convert solar power and water into biofuel. As such, production of hydrogen from water utilizing solar energy is regarded as a far more environmentally benign process compared to related chemical processes. Such is the interest displayed in using these enzymes that several attempts have been made to understand, tune the catalytic bias, and develop their synthetic homologs [6,7,8,9,10,11,12,13,14,15,16,17,18].

At present, cyanobacteria and green algae are considered optimal candidates for hydrogen evolution, being the only known organisms that can both express functional hydrogenase and perform oxygenic photosynthesis [4,19,20]. The two organisms possess two distinct classes of hydrogenase; [NiFe] and [FeFe] hydrogenase respectively. A variety of organisms possess [NiFe] hydrogenases, however, [FeFe] hydrogenases are typically present only in algal species and a few prokaryotes. They are completely excluded from all cyanobacteria examined to date [21]. Though the two hydrogenases essentially catalyze the same reaction, they are structurally distinct and phylogenetically unrelated. The high turnover [FeFe] hydrogenases are known to be extremely oxygen labile [21]. On the other hand, cyanobacteria possess [NiFe] hydrogenases that are only reversibly inhibited by molecular oxygen, and some others, produced in aerotolerant organisms such as *Aquifex aeolicus*, are able to function in the presence of oxygen, a characteristic not known of [FeFe] hydrogenase [17,21,22,23].

Over the last few decades, cyanobacteria have been surveyed for hydrogen production. Depending on their physiology and structure, the different divisions of cyanobacteria are known to evolve hydrogen through different mechanisms [24,25,26,27]. For most unicellular, non-heterocystous and filamentous cyanobacteria, nitrogen fixation and photosynthesis occur in the same cell. In these cyanobacteria, the partition between photosynthetic oxygen evolution and hydrogenase/nitrogenase mediated hydrogen production is achieved through temporal separation as exemplified by unicellular *Synechococcus* PCC 7942 and non-heterocystous filamentous *Spirulina* sp. [24,25,26,28,29]. Other filamentous, non-heterocystous cyanobacteria rely on the development of larger colonies to create internal micro-anaerobic conditions such that certain internal filaments develop the capacity to fix nitrogen and produce hydrogen while the surrounding cells act as a sheath [30]. Phylogenetically more advanced, heterocystous, cyanobacteria such as *Nostoc* have evolved spatial separation in the form of micro anaerobic chambers for the expression of the oxygen-sensitive enzymes [24,25,26,31].

Cyanobacteria primarily possess three enzymes related to hydrogen production including the bidirectional Hox enzyme that catalyzes both hydrogen oxidation and proton reduction; the nitrogenase enzyme complex that produces hydrogen as a byproduct of nitrogen fixation, and the uptake hydrogenase that functions to oxidize hydrogen and is found closely associated with the nitrogenase complex [24,25,26,27,32]. The precise physiological role of the bidirectional Hox enzyme is still under debate. It is believed to function as an electron valve to release any excess electrons produced under photosynthetic or fermentative conditions [16,33,34,35,36]. In cyanobacteria, the uptake enzyme is found present in all nitrogen fixing species with the exception of the nitrogen fixing unicellular strain *Synechococcus* BG04351 and some *Chroococcidiopsis* isolates [37]. At the same time, the uptake hydrogenase has never been identified in a non-nitrogen fixing strain. The functions of the uptake hydrogenase are well characterized. Likely, it serves three functions including (i) the removal of oxygen from the nitrogenase site via the knall gas reaction (ii) to regain ATP consumed during the nitrogen fixation reaction and (iii) to prevent feedback inhibition to nitrogenase created by the buildup of hydrogen, particularly in the heterocysts [26]. In addition, hydrogen oxidation may provide additional reductant for nitrogen fixation, photosynthesis, and other reductive processes. There is as yet no evidence of any hydrogen sensory hydrogenases encoded by e.g., *hupUV* in cyanobacteria. Also, the cyanobacterial hydrogenases are devoid of [NiFeSe] type, commonly found in some anaerobic bacteria [25].

Whereas most of the characterized [NiFe] hydrogenases are hydrogen oxidizing, the cyanobacterial bidirectional Hox hydrogenases are interesting in terms of their bidirectional catalytic property [17]. Protein film electrochemistry has shown that the bidirectional Hox hydrogenase from *Synechocystis* PCC 6803 is “moderately” biased toward proton reduction as opposed to hydrogen oxidation [17]. This unusual catalytic adaptation may be linked to its role in maintaining the cellular redox potential as the organism swings from dark to light conditions [17]. The Hox enzyme from cyanobacteria has been studied in detail at the molecular level including its interacting partners and redox regulation [6,33,38,39]. In cyanobacteria, the enzyme is pentameric encoded by *hoxEFUYH*. The small and large catalytic subunit is encoded by *hoxY* and *hoxH* respectively, whereas *hoxEFU* encodes the diaphorase moiety. The physical location of the five structural genes differs from one cyanobacterium to the next [24,33,40,41]. In some cyanobacteria, they appear as a single operon while in others they are clustered in one part of the chromosome but interspersed with open reading frames (ORFs) at different positions, while in other organisms they are distributed throughout the genome [24,25]. The HoxH harbors the active metal center containing nickel and iron that are held in close proximity by two disulfide bridges that are provided by two cysteine residues. Additionally, the iron is coordinated by two cyanide ions and one carbon monoxide, whereas the nickel ion is coordinated by two additional cysteine residues [42,43,44,45]. For coordination, cysteine residue was found critical and mostly conserved across this family of enzymes [21,22]. The biosynthesis of such an enzyme is also a complex process and requires the help of several additional proteins (HypABCDEF) to incorporate the FeS clusters within the small subunit and metal ions and CO and CN ligands into the active center [21,46,47]. For maturation, HoxH (the large subunit) of the bidirectional hydrogenase undergoes specific endopeptidase cleavage at the *C*-terminus catalyzed by HoxW [6,21,46,48,49]. The *hypABCDEF* genes involved in this process have been identified in *E. coli* and it is known that their homologs are present in all organisms possessing [NiFe] hydrogenases. Within the cyanobacteria group, the maturation of the [NiFe] hydrogenases has not been studied in detail; however, several genes involved in this process have been identified clustered or scattered throughout the genomes [25,46,50,51,52]. LexA and two members of the AbrB-like family appear to be involved in the transcriptional regulation of the cyanobacterial Hox hydrogenase [6,53,54,55,56].

In this review, recent advancements that have a significant impact in the field of bidirectional Hox hydrogenase research are discussed. In addition, towards the development of more robust hydrogen-producing cellular factories, the feasibility of expression of non-native [NiFe] and [FeFe] hydrogenase in cyanobacteria are also summarized.

## 2. Key Advances in Cyanobacterial Research

The bidirectional Hox hydrogenase is the key [NiFe] hydrogenase homolog involved in hydrogen production in cyanobacteria. Section 2.1 discusses the recent work that established ferredoxin as the natural electron donor to the bidirectional Hox hydrogenase in place of the generally accepted NAD(P)H. In Section 2.2, the expression and limitations of non-native hydrogenases including but not restricted to [NiFe] hydrogenase are discussed. Studies suggesting base pair substitutions in FeS cluster binding motifs to improve the efficacy of non-native [NiFe] hydrogenase are highlighted in Section 2.3.

### 2.1. Hox Reduced by NAD(P)H/NADH in Synechocystis—A Myth Busted

*Synechocystis* PCC 6803 (hereafter *Synechocystis*) is a hydrogen producing strain that has been studied for several decades. It is a non-nitrogen fixing, genetically tractable, photosynthetic model organism. Recent protein film electrochemistry studies have demonstrated that the bidirectional Hox hydrogenase in *Synechocystis* has a bias towards proton reduction as opposed to hydrogen oxidation [17]. The physiological role of this enzyme is bidirectional. It acts as a valve to both “release” the excess electron generated under anoxic conditions, as molecular hydrogen, and also to “generate” electrons through hydrogen oxidation. Hydrogen production serves to dissipate the excess electron load derived either from fermentative metabolism in the dark or from the photosynthetic electron transport chain obtained during the swing from dark anaerobic to aerobic photosynthetic conditions [16,34,35]. Even though hydrogen production from the bidirectional Hox hydrogenase in *Synechocystis* has been studied for a long time, to date there are still several gaps in our understanding of the cyanobacterial hydrogen production process. With their unusual catalytic bias and the ability to be quickly reactivated after aerobic inactivation, bidirectional Hox hydrogenase can have immense application in bio-based technologies [57,58,59]. However, understanding the cellular metabolomics, in relation to hydrogen production is critical to establishing a photobiological hydrogen based industry [16,34,35].

The very discovery of the bidirectional Hox hydrogenase was shrouded in controversy for a long time. Only after extensive debate, did Houchins and Burris clearly show the presence of another hydrogenase in addition to the uptake hydrogenase in nitrogen-fixing cyanobacteria [60,61]. This reversible hydrogenase that catalyzed both hydrogen oxidation and proton reduction was isolated for the first time from the crude cell extracts of *Anabaena* PCC 7120 [62]. In *Synechocystis*, hydrogen production is associated with a single enzyme called the bidirectional Hox hydrogenase belonging to the [NiFe] class of hydrogenases [34]. However, the functional purpose of the constitutively expressed bidirectional Hox hydrogenase in aerobic photosynthetic *Synechocystis* remains elusive and is a debated topic. In an attempt to answer the question, the *hox* gene transcripts were studied under different physiological conditions [39]. It is predominantly thought to act under anaerobic conditions either to consume hydrogen to prevent the loss of reductant from the cell or during fermentation to dispose of the excess reductant as molecular hydrogen [2,16,34,35]. The role of the bidirectional Hox under aerobic condition is still unclear. Despite its aerobic expression, it is not oxygen-tolerant. Aerobic inactivation is total and nearly instantaneous, however, it is quickly (<90 s) reactivated by removal of oxygen and exposure to reducing conditions [17,22,63,64]. Moreover, studies showed that the cell coped well even in the absence of this enzyme, as was apparent in the *hoxYH* gene deletion mutant that showed no distinguished phenotype [65]. Comparative proteomic studies of the mutant and the wild type, grown under identical conditions, revealed differential fold changes for only 17 of the 210 identified proteins. Most of these proteins were related to the redox and energy state of the cell. The Hox deletion strain thus exhibited plasticity and metabolic adaptability [65].

Research in this field spans several decades yet there are several aspects, in this area of research, that still remain confounding. The cellular location of the Hox hydrogenase and its interaction with the photosynthetic electron transport chain remained contentious for a long time. In contrast to the uptake hydrogenase, the bidirectional hydrogenase is soluble after breaking the cyanobacterial cells. Immunological and membrane solubilization studies indicated its possible association with the cytoplasmic membranes or the thylakoids [34]. It was known that the hydrogenase lacks a membrane-spanning domain, thus indicating a possible interaction with an integral thylakoid membrane complex [25,38]. Only a very recent study involving green fluorescent protein tagging of the HoxF diaphorase subunit clearly indicated uniform dispersal of the hydrogenase through the thylakoid membranes and also a “subpopulation” localized to the discrete puncta in the distal thylakoid [66]. The HoxH and HoxY subunit association to the thylakoids was confirmed by immunogold electron microscopy [66].

Other issues that need to be resolved in *Synechocystis*, and also in other cyanobacteria in general, include the determination of factors that influence the partitioning of the reducing power between respiration and photosynthesis that share the cytochrome b_6_f complex (respiratory complex III) Recently, enhanced green fluorescent tagging and confocal fluorescence microscopy imaging in live cells was used to explore the factors that might control the prevalence of respiratory electron transfer to terminal oxidases *vs.* photosystem I (PSI) in the cyanobacterium *Synechococcus elongates* PCC 7942 [67]. The authors investigated the distribution of two key respiratory electron donors, type-I NAD(P)H dehydrogenase (NDH-1) and succinate dehydrogenase (SDH) in submicron scales (100–300 nm) under high to low light regimes and also in the presence of electron transport inhibitors. Their study indicated that complex redistribution influenced the partition of reducing power. However, the physical factors that controlled the distribution of the respiratory complexes within cyanobacterial thylakoid membranes were deemed as subjects for future investigation [67].

Another issue centers around the involvement of the HoxEFU diaphorase subunits in NAD(P)H-dehydrogenase complex I (NDH-I) of the respiratory chain. In cyanobacteria NDH-I was known to be comprised of 11 subunits whereas in all other organisms the complex was comprised of at least 14 subunits [41,51,62,68,69,70,71]. Subsequent proteomic analyses of the NDH-1 complexes from *Synechocystis* revealed the presence of several additional novel subunits including NDH-1M, NDH-1N, and NDH-1O besides carbon uptake subunits [71,72,73]. The diaphorose moiety of the bidirectional Hox hydrogenase, *hoxEFU*, bears homology to NADPH. It was therefore suggested that the “three missing genes” were encoded by the Hox diaphorase moiety and were simultaneously employed by the reversible Hox hydrogenase and the NDH-I complex [41,68,69,74]. However, there are two issues with this suggestion. Firstly, Hox does not occur ubiquitously in all cyanobacteria, for instance, *Nostoc* PCC 73102 has no bidirectional hydrogenase activity but respires with rates comparable to those of other cyanobacteria [75]. Secondly, engineered strains with a mutation in either *hoxF* or *hoxU* did not show any bidirectional hydrogenase activity but still retained unimpaired respiratory activity [76]. In this light, Marreiros *et al.* [77] recently proposed an alternative perspective suggesting that the homology may be based on the evolutionary origins of complex I.

In addition to the above is another fundamental question regarding the possible electron donor to these hydrogenases. After much debate, it was established that NAD(P)H is the electron donor to the bidirectional Hox hydrogenase in *Synechocystis*. Although this was well accepted, there was always an element of surprise associated with this fact. The pyridine binding subunits of the diaphorase moiety have to date not been established [74]. In the text below, we describe why NAD(P)H was earlier accepted as the electron donor and more recently why ferredoxin was suggested as a more reasonable choice.

Sequence alignment of Hox hydrogenase from several different cyanobacteria showed a high similarity to the soluble NAD-reducing hydrogenase of *Alcaligenes eutrophus* [74]. This was the first evidence pointing towards NADPH as a direct redox partner to the [NiFe] homolog of cyanobacteria. Early activity experiments conducted on *Anacystis nidulans* bidirectional enzyme clearly indicated NADPH as the preferred substrate [69]. NADPH was later confirmed as an electron donor to the bidirectional Hox hydrogenase in studies using *Synechocystis* M55 mutant deficient in respiratory complex NDH-I. The mutant depicted high, sustained hydrogen production activity as compared to the wild type [35]. The paper rationalized suppression of the NADPH-consuming NDH-1 enzyme for the increased activity of the NADPH-dependent, reversible Hox hydrogenase. In cyanobacterial cells the redox status of the NADPH/NADP^+^ pools is controlled by the activities of photosynthesis and respiration [35]. NADPH is also the substrate for the respiratory complex NDH-I encoded by the *ndh* genes. The developed M55 mutant was devoid of the *ndhB* genes. This likely led to large reserves of reduced NADPH pools. The authors discussed that under anoxia, the reduced NADPH pools may be consumed by the bidirectional Hox hydrogenase [35]. Contrarily, studies on HoxE deletion strain suggested better performance of NADH over NADPH, as electron donors to the bidirectional Hox hydrogenase in *Synechocystis* [78]. H/D exchange rates conducted on soluble wild type cell extracts indicated that NADH was a much better electron donor compared to NADPH, however for the HoxE deletion mutant, supplementation of the soluble extract with NADPH showed higher activity. H/D exchange rates determine the turnover of the catalytic site independently of the electron transfer. This led the authors to conclude that whereas NADH-mediated interaction occurred via the diaphorase moiety, the NADPH interaction occurred through an alternate mechanism [78]. Although it was generally accepted that the pyridine nucleotides were involved in transport of electrons to the Hox hydrogenase, there was general confusion regarding the nature; NADPH or NADH. While NADH is a product of glycolysis, NADPH is a product of the photosynthetic electron transport chain. Nevertheless, by the late 1990s and early 2000 it was well established that NAD(P)H was the possible redox donor to the bidirectional Hox hydrogenase. This was then considered a milestone discovery in relation to cyanobacterial hydrogen production [24]. Although well established the thermodynamic limitation of such a reaction was always debated. 

With the identification of the electron donor, the hydrogen production pathway in *Synechocystis* was established [79]. It was suggested that in darkness and under anaerobic conditions, the reductant was generated either from fermentation (NADH from glycolysis) or from catabolism of the stored glycogen (NADPH), derived from the pentose phosphate pathway. It was accepted that these reductants were channeled through the pyruvate:ferredoxin oxidoreductase (PFOR)-ferredoxin-FNR-NAD(P)H to the Hox hydrogenase and coupled with cytoplasmic proton to produce molecular hydrogen [79,80,81]. Under light preceded by darkness, a short burst of hydrogen was observed. This was governed by ferredoxin-NADP^+^ reductase (FNR)-hydrogenase [34,35]. The extent of hydrogen evolution was known to be governed by the redox state of the NAD(P)H pools controlled by the activities of photosynthesis and respiration [34,35]. Owing to the electrochemical potential difference of about, −320 mV for the NADP^+^/NADPH couple and −420 mV for H^+^/H_2_, the thermodynamic limitations of such a system remained questionable. Under such limitations, hydrogen production was possible only in the presence of very high, up to 99.9%, reduced NADP^+^ pool. However, practically, it does not seem feasible for the cells to possess such a high ratio of NADPH/NADP^+^ [16].

A case in point is the hydrogen production measurements from the cyanobacterium *Lyngbya* that can be as high as 450 µM [82]. Calculations by Gutekunst *et al.* [16] suggest a NADH/NAD^+^ ratio of 1000 would be required to support such high concentrations of hydrogen. The authors cited a redox ratio of 0.03 in *E. coli* under heterotrophic conditions [16,83,84]. The same or similar conditions may be extrapolated to cyanobacteria and thus the presence of a redox ratio of 1000 appears unrealistic [16]. Moreover, Gutekunst *et al*. [16] suggested that assays in which NAD(P)H was regarded as the electron donor used high concentrations between 12 µM to 0.3 mM [16,35,78]. Such high concentrations cannot be accounted for in the living cell [16]. In addition, lately it was shown that NADPH was a poor electron donor as compared to NADH [16,78]. This was in contradiction to the earlier propagated role of the bidirectional Hox hydrogenase as an electron sink for the disposal of surplus electrons coming from PSI via ferredoxin, FNR and NADPH.

In view of the above, alternate molecules that could serve as redox acceptors or donors to the bidirectional Hox hydrogenase were surveyed [16,78]. Important limitations of the studies conducted so far appear to be lack of any *in vivo* analysis. It was observed that upon elution the pentameric HoxEFUYH hydrogenases tends to dissociate into the NAD(P)H binding diaphorase unit HoxEFU and the active hydrogenase HoxYH. Often *in vitro* and *in vivo* results have thus shown discrepancy. HoxE deletion mutants were devoid of hydrogen production *in vivo* however, *in vitro*, on addition of methyl viologen hydrogen production could still be detected [38,78]. Indeed, this shows that such small molecules have the potential to bypass the diaphorase moiety to donate electrons directly to the active site.

**Figure 1:**
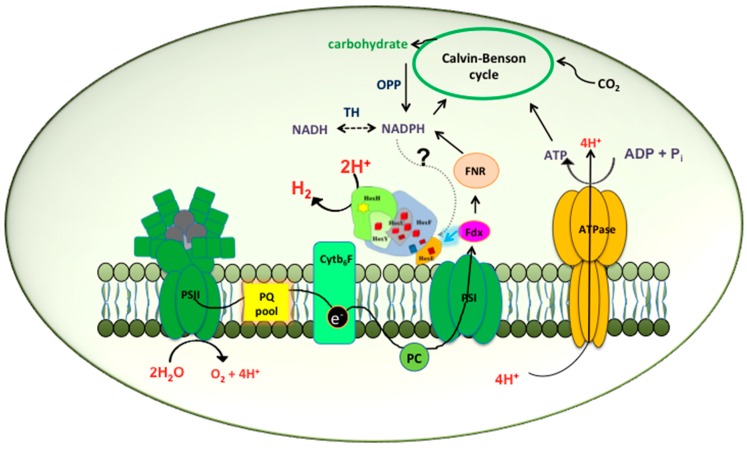
Biochemistry of hydrogen production in *Synechocystis* PCC 6803 by direct biophotolysis. In this pathway the reducing equivalents are obtained directly from the splitting of water at PSII. The electrons are transferred into the photosynthetic electron transport chain through a series of transport molecules including plastoquinone (PQ), cytochromeb_6_F (Cyt b_6_f) and plastocyanin (PC), and move through photosystem I (PSI) to reduce ferredoxin (Fdx) which goes on to reduce NADP^+^ to NADPH via the enzyme ferredoxin NADP^+^ reductase (FNR). At the same, time the reduced ferredoxin also has the capacity to directly donate the electrons to the Hox hydrogenase indicated by the highlighted arrow. Under the present conditions hydrogenase can compete with the Calvin cycle for hydrogen production till it is inactivated due to the evolution of oxygen at PSII. The dotted arrow speculates NADPH as another possible electron donor to the hydrogenase. The dotted double-headed arrow under TH suggests a hypothetical conversion of NADPH into NADH and *vice versa*. Abbreviations: OPP: Oxidative Pentose Phosphate pathway; TH: transhydrogenase; ATP: adenosine triphosphate; ADP: adenosine diphosphate.

Recently, Gutekunst *et al.* [16] suggested modifications in the reported mechanism of hydrogen production from *Synechocystis*. Detailed experiments were carried out both *in vivo* and *in vitro* to determine the redox partner to the bidirectional hydrogenase in *Synechocystis*. While carrying out *in vitro* assays, they found that the purified holoenzyme was not intact and incorporated only sub-stoichiometric levels of HoxE [16]. Earlier experiments had established the importance of HoxE subunit in the mediation of the pyridine nucleotides to hydrogenase [38,85]. An incomplete holoenzyme had the possibility to skew the results [85]. Therefore, the authors carried out studies on whole cell extracts [16]. Concentrations of 1.5 mM NADPH failed to induce any measureable hydrogen production in the cell-free extracts, however, related amounts of NADH led to the production of low levels of hydrogen [16]. Concurrently, addition of *in situ* concentrations of flavodoxin (Flv) or ferredoxin (Fd) led to the production of high rates of hydrogen. Ferredoxins are small, mostly acidic iron sulfur cluster containing proteins that usually possess a highly negative redox potential and act as electron donors in various metabolic pathways [86]. The authors did not observe a strict requirement for NADH to stimulate the electron transfer by ferredoxin. The results were further authenticated by the development of merodiploid FNR mutants that demonstrated prolonged hydrogen production capacity in keeping with the observation that ferredoxin is the direct electron donor to the hydrogenase [16] (Figure 1). If not, the FNR deletion mutant was likely to disrupt the electron transfer between the Fd-FNR-NADPH and the hydrogenase resulting in decreased hydrogen production [16]. Also, the thermodynamics of such a system corresponded well since the mid-point reduction potential of Fd/Flv is −440 mV compared to −420 mV of the H_2_/H^+^ redox couple [16].

**Figure 2:**
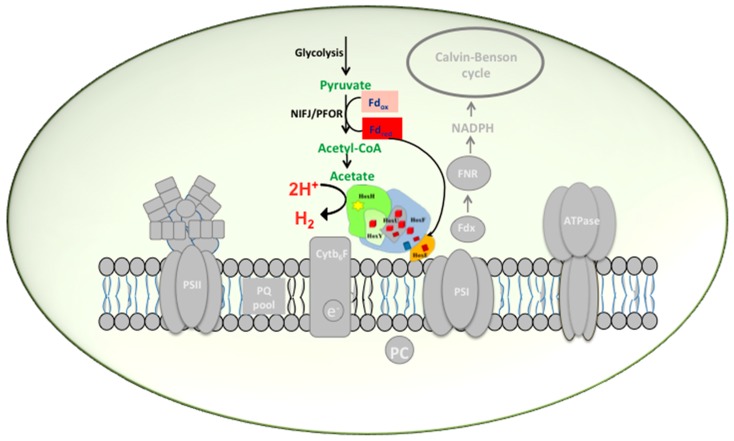
Biochemistry of hydrogen production in *Synechocystis* PCC 6803 by indirect biophotolysis. In darkness, under anaerobiosis, when the PSII is inactivated, glycogen is catabolized to pyruvate by glycolysis. Pyruvate is further oxidized to acetyl CoA during which ferredoxin is reduced by pyruvate formate oxidoreductase (PFOR). Reduced ferredoxin has the capacity to directly donate electrons to the Hox hydrogenase.

One of the proposed functions of hydrogenase in *Synechocystis* is to maintain the redox balance under dark, anaerobic and fermentative conditions *en route* PFOR-ferredoxin-FNR-NADPH [16,33,34,35,36,79,80,81]. However, delta FNR mutants failed to show impaired hydrogen evolving capacity under fermentative conditions [16]. In contradiction, pyruvate formate oxidoreductase (PFOR) deletion mutant showed severely impaired hydrogen production capacity [16]. This together with the fact that NADPH is an uncommon electron donor under fermentative conditions inclines towards the acceptance of ferredoxin as the most likely electron donor under these conditions (Figure 2). Identification of amino acid residues that play a vital role in Hox-ferredoxin interaction further support this inference. Certainly further investigations are also required to understand the mechanisms by which electrons are partitioned between the various pathways in which ferredoxin plays a central role.

Recently, Burroughs *et al.* [66] addressed the issue of bidirectional Hox hydrogenase localization in *Synechocystis*. They evidenced thylakoid membrane association of the HoxE subunit of the pentameric hydrogenase. This association also implied access to a membrane localized donor for electron transport either during fermentation or whilst coupling to the photosystem electron pathway [66]. Consistent with the finding of Gutekunst *et al.* [56], their data suggested the presence of a reduced form of flavodoxin and ferredoxin near the thylakoids under conditions favorable for hydrogen production. The authors suggested that thylakoid membrane association positioned the Hox hydrogenase vicinal to the reduced Fd. This provided easy accessibility to its reduced pool [66].

Though the recent finding established ferredoxin as a redox partner, the possibility of NAD(P)H further assisting the process cannot be completely overlooked. It may be an option that under certain conditions, NAD(P)H may induce low rate hydrogen production. This can be argued in the light of the previous reports where hydrogen production was observed in cell free extracts using high concentrations of NAD(P)H [35,70,78]. Moreover, *Synechocystis* strain M55, showed compelling evidence of sustained Hox activity, a direct reflection of the utilization of the accumulated NADPH reserve pool [35].

This recent insight that ferredoxin and not NAD(P)H acts as a likely electron donor to the bidirectional Hox hydrogenase has far reaching implications for the biofuel industry. More importantly, the knowledge was established in *Synechocystis*, an organism favored for hydrogen production due to the ease of amenability to genetic manipulation. This insight also sets the path for determining a plausible ferredoxin-mediated interaction with the bidirectional Hox hydrogenase in other cyanobacteria.

Light driven hydrogen energy has been epitomized as an alternative way to generate the energy in an environmental friendly manner. In regard to continuous hydrogen production, one of the most ambitious visions of the biofuel industry is to drive photobiological hydrogen production by direct coupling of linear electron transport chain, starting with the solar driven water splitting at PSII [87]. However, one long-standing criticism of such a process involves the low turnover and hydrogen yields from the homologs of [NiFe] hydrogenases. Addressing this issue, replacement of the native cyanobacterial [NiFe] hydrogenases with foreign, more catalytically efficient hydrogenases has recently been accomplished.

### 2.2. Introduction of Non-Native Hydrogenases in Cyanobacteria

Expression of non-native hydrogenases has been widely deemed as one of the ways to improve hydrogen production from cyanobacteria. These include the high turnover [FeFe] hydrogenase and some oxygen and thermo-tolerant [NiFe] hydrogenases. Efforts in this direction have been ongoing for a long time. Technical hurdles had stalled the process till the advent of synthetic biology techniques. Thereafter, four successive publications appeared between 2011 and 2012 (Table 1). In this league, the first publication appeared by Ducat *et al*. [88] demonstrated the feasibility of expression of an [FeFe] hydrogenase from *Clostridium acetobutylicum* into *Synechococcus elongatus* PCC 7942. Both, *in vitro* and *in vivo* activity of the non-endogenous hydrogenase was demonstrated.

**Table 1:** Studies on the expression of non-native hydrogenase in cyanobacteria.

Organism	Genes Expressed	Comments	Reference
*Synechococcus elongatus*	Expression of [FeFe] hydrogenase and the accessory HydEFG from *Clostridium acetobutylicum* in neutral site 3 and 1 respectively.	The first report on expression of non-native [FeFe] hydrogenase along with their accessory genes in cyanobacteria. The article clearly demonstrated *in vitro* and *in vivo* hydrogenase activity. The authors established that the *in vivo* hydrogenase activity was connected to the light-dependent reactions of the electron transport chain.	[88]
*Synechocystis* PCC 6803	Expression of [FeFe] *hydA* from *Chlamydomonas reinhardtii* in a non-coding region of the *Synechocystis* chromosome(s) identified between the *sll1865* and *sll1864* genes, which encode for the peptide chain release factor 2 and for a chloride channel protein, respectively.	The report suggested that foreign [FeFe] hydrogenase could be matured in absence of auxiliary proteins, HydEFG. The report showed five times higher hydrogen production from the recombinant cells. This however, needs further confirmation.	[89]
*Anabaena* PCC 7120	Expression of *Shewanella oneidensis* MR-1 [FeFe] hydrogenase and accessory genes on a pK3 self-replicating vector.	The first publication that showed the expression of an [FeFe] hydrogenase under a heterocyst specific promoter, in a filamentous organism. [FeFe] hydrogenase expression was detected and the protein was purified from aerobically grown filaments cultivated under nitrate-depleted conditions. Activity assays confirmed *in vitro* and *in situ* activities, however, *in vivo* activities could not be detected. This perhaps demonstrated substrate limitation for the successful endogenous activity of the introduced hydrogenase.	[90]
*Synechococcus elongatus*	Expression of [NiFe] hydrogenase from *Alteromonas macleodii* and *Thiocapsa roseopersici*.	The first report that demonstrated expression of non-native [NiFe] hydrogenase in cyanobacteria. *Thiocapsa roseopersicina* (*hynSL*) and the 11 accessory genes from *Alteromonas macleodii* were co-expressed. The article showed *in vitro* activity of the expressed protein.	[91]

The authors not only expressed the structural [FeFe] hydrogenase but also the accessory operon genes including *hydE*, *hydF* and *hydG*. The accessory operon is required for maturation, assembly and insertion of the di-iron subcluster into the active site of [FeFe] hydrogenases to form the complete six-iron prosthetic group (H cluster) and the catalytically active protein [92,93]. Whereas the accessory genes were placed in the neutral site I under the regulation of the constitutive *psbA2* promoter, the structural *hydA* gene was placed in neutral site II under the regulation of the IPTG induced *lac* promoter. Hydrogen production was studied under both anoxic light and dark conditions. Under anoxic conditions, the carbohydrate reserves are broken down and the reducing equivalents thus generated can be disposed of by the [FeFe] hydrogenase. Under light, photo-fermentation occurs, the electrons re-enter the cyclic PSI, are re-energized and subsequently delivered to hydrogenase via ferredoxin. For functional expression of the oxygen sensitive hydrogenase, PSII was inactivated by the addition of DCMU (3-(3,4-dichloro-phenyl)-1,1-dimethylurea). DCMU is an inhibitor of photosynthesis. It blocks the plastoquinone-binding site of photosystem II, and thus disrupts the electron flow from photosystem II to plastoquinone. This interrupts the linear photosynthetic electron transport chain and the ability to split water and generate oxygen. In the dark, the electrons from the fermentation of carbohydrate are directly supplied to the hydrogenase via PFOR-ferredoxin [16]. Ducat *et al*. [88] observed higher hydrogen production under light while it was greatly reduced in the dark or in the presence of DBMIB (2,5-dibromo-3-methyl-6-isopropyl-*p*-benzoquinone), an inhibitor of cyt b_6_f complex [88]. Linking a clostridial ferredoxin to *HydA* further increased the fermentative hydrogen production capacity by facilitating the development of an insulated circuit for transfer of electrons to the hydrogenase. The authors showed more than 500-fold increase in hydrogen production as compared to the wild type. At the same time little or no hydrogen was observed in the wild type cells harboring the native circadian controlled bidirectional [NiFe] hydrogenase. However, the authors did not study the effect of nitrogen deprivation in the dark. Since nitrate metabolism consumes a significant flow of reductants, nitrate starving can enhance hydrogen production [33,36,94]. It is interesting to note that the non-endogenous genes were expressed in the wild type background in the presence of native bidirectional Hox hydrogenase. This indicated that substrate was not limiting, as previously speculated. In a contradictory publication Berto *et al.* [89] expressed a functional [FeFe] hydrogenase from the green algae *Chlamydomonas reinhardtii* in *Synechocystis* PCC 6803. They showed functional activity of the hydrogenase in the absence of the related accessory genes *hydE*, *hydF* and *hydG* [89]. This study, however, needs to be confirmed.

The ultimate aim of using cyanobacteria is to co-harness the solar energy for simultaneous photosynthesis and renewable energy production. The direct production of hydrogen circumvents the inefficient Calvin cycle and offers the prospect of highly efficient fuel production. However, the evolution of oxygen at PSII appears a challenge for the expression of oxygen labile hydrogenase. In the presence of oxygen the di-iron site of [FeFe] hydrogenase is attacked and rendered inactive. Stripp *et al.* [95] propounded two possible theories to explain this inactivation. The first theory suggested that oxygen reacts with the 2Fe domain to form a reactive oxygen species that migrates the short distance to attack the [4Fe4S] cluster. The second theory suggested that a superoxide is formed by reaction at presumably the distal Fe of the di-iron site of [FeFe] hydrogenase. This however, remains bound as a strong oxidant, inflicting long range oxidation of the [4Fe4S] cluster by through-bond electron transfer. It was suggested that the oxygen attack on the [4Fe4S] cluster could be prevented by steric and thermodynamic limitations [95]. To overcome the disruption of the active site, nature has propounded various ways such as temporal and spatial separation of the anoxic hydrogen production process and the oxygenic photosynthetic splitting of water at PSII.

In the third article published in this area of research, Gartner *et al.* [90] attempted to demonstrate the spatial separation of the [FeFe] hydrogenase by expressing them in micro anaerobic cellular compartments known as heterocysts [90]. Heterocysts maintain an oxygen-depleted interior due to an inactive PSII, high respiratory quotient and the presence of a specialized cell envelope [96,97]. Although nitrogenase is the primary hydrogen producer in such organisms, attempts to modify the nitrogenases to enhance the hydrogen production, have received only limited success to date [32,98]. On the other hand, [FeFe] hydrogenases have turnover numbers, 1000-fold greater than those of nitrogenases, therefore their expression was speculated to increase the hydrogen yields several fold compared to the wild type enzymes. The [FeFe] hydrogenase from *Shewanella oneidensis* MR-1 was introduced and expressed in *Anabaena* PCC 7120 using the heterocyst-specific promoter *PhetN*. The choice of the promoter remained crucial in this study allowing transcription and translation only under nitrate deprived pro-heterocyst formation conditions. The presence of an active PSI ensured the availability of low potential electrons and a reduced ferredoxin that could potentially be linked to the non-endogenous hydrogenase. Both, *in vitro* purified enzyme activity and *in situ* whole cell enzyme activity was observed using methyl viologen as a promiscuous electron donor, however, the authors did not observe any *in vivo* enzyme activity. This may not be a complete surprise as addition of methyl viologen has the potential to donate electrons directly to the active site. *In vivo* hydrogen production may have been limiting due to two possibilities. Primarily, the availability of electrons may be impeding the enzymatic activity. Secondly, *Shewanella* [FeFe] hydrogenase is dimeric and is known to interact with cytochrome c instead of ferredoxin. Therefore, possibly, *in vivo* hydrogen production failed to occur due to lack of interaction with the appropriate electron donor. To conclude, Gartner *et al.* [90] were the first to report expression of [FeFe] hydrogenase in filamentous cyanobacteria, though *in vivo* activity of the enzyme could not be determined [90]. Development of such a system could ensure continuous production of hydrogen which could bypass the inefficiencies associated with energy transfer in unicellular cyanobacteria. This may eventually lead to the development of more sustainable hydrogen energy systems.

In addition to [FeFe] hydrogenases, attempts have been made to introduce non-endogenous [NiFe] hydrogenase into cyanobacteria. Weyman *et al*. [98] expressed [NiFe] hydrogenases from *Alteromonas macleodii* (*A. macleodii*) and *Thiocapsa roseopersicina* (*T. roseopersicina*) in *Synechococcus elongatus* [98]. The advantage of using [NiFe] homolog over the [FeFe] hydrogenases was their increased half-life to temperature and oxygen stress [99]. Expression of such oxygen tolerant hydrogenases in photosynthetic systems may open up new avenues in cyanobacterial hydrogen production. However, the expression of such a hydrogenase in cyanobacteria was challenging. *A. macleodii* hydrogenase is comprised of two structural genes comprising the small and large hydrogenase subunits, encoded by *hynSL* respectively. The structural genes are surrounded by eight other genes (*hynD*, *hupH* and *hypCABDFE*) known to be involved in hydrogenase maturation. In accordance, the authors cloned a 13 kb fragment including the *A. macleodii* structural genes (*hynSL*) and 11 adjacent hypothetical accessory genes in an IPTG-inducible expression vector. The vector was transformed into an *Escherichia coli* (*E. coli*) mutant strain lacking its native hydrogenases [100]. Upon induction, HynSL from *A. macleodii* expressed in *E. coli* and was active, as determined by the *in vitro* hydrogen evolution assays. The HynSL from *A. macleodii* shares about 60% identity to the HynSL from *T. roseopersicina* [100]. The authors also showed successful complementation of the *T. roseopersicina* structural gene with the accessory gene cluster from *A. macleodii*.

The functional constructs from *E. coli* were also expressed in *Synechococcus elongates* (*S. elongates*) [98]. The structural and accessory genes were driven by Ptrc, one of the strongest inducible promoters for expression in cyanobacteria. *In vitro* hydrogen production was studied from recombinant strains expressing the *A. macleodii* hydrogenase operon together with its accessory gene cluster in neutral site I of *S. elongatus* RC41strain lacking the native HoxYH. Disappointing hydrogen production rates of only one tenth of that produced by the wild type *S. elongatus* were observed. Complementing the *T. roseopersicina* hydrogenase structural gene with the accessory genes from *A. macleodii* resulted in even lower hydrogen yields [98]. These low yields reflect the complexity of the system. Clearly, there exist other limiting factors that need to be addressed before such a system becomes viable. A critical drawback of the study includes the lack of any *in vivo* data analysis. This would have demonstrated a better understanding of the linking of these hydrogenases to the native microbial metabolism.

It is to be noted that in all the above three studies, the hydrogen operons were cloned in neutral sites 1 or 2. Neutral sites have been identified on the basis that their loci can be disrupted without display of any aberrant phenotypes [101]. However, a more careful recent study revealed that several constitutive promoters from *E. coli* behave in a circadian manner when expressed in these neutral sites [101]. This observation points towards a circadian influence over neutral sites 1 and 2. A case in point is the study of a constitutive conIIp constitutive promoter that resulted in rhythmic expression profile of *luxAB* reporter when expressed from NSI and NSII in *S. elongatus* [101]. Xu *et al.* [101] for the first time overexpressed the circadian controllers KaiA or KaiC that could reprogram the circadian cycle such as to positively influence the expression profile of endogenous and non-endogenous genes including those useful for the production of biofuels. Accordingly, expression of KaiA in *Synechococcus* RC41 strain lacking native bidirectional Hox hydrogenase but incorporating the *A. macleodii* hydrogenase improved the expression of HynL and increased hydrogen production two-fold. Though, this improvement failed to bear any significant implication, as the total yield was still lower than the wild type, this still revealed an important breakthrough in overcoming the limitations of expression in cyanobacteria. Clearly, the study demonstrated the influence of the cell’s endogenous clock upon the expression of exogenous products [101].

Thus, out of all the published reports on non-endogenous hydrogenase expression in cyanobacteria only one reported successful *in vivo* activity. These results hint towards other possible limitations in the system including but not restricted to internal electron reserves that should be channeled towards hydrogenase.

### 2.3. Introduction of “Improved” Non-Native Pathways into Cyanobacteria

Different strategies have been used to improve the hydrogen production capacity of photosynthetic organisms [19,20,32]. The introduction of non-native pathways has been explored either by expression of a catalytically efficient [FeFe] hydrogenase or a comparatively more oxygen and thermo-tolerant [NiFe] homolog of *A. macleodii* HynSL hydrogenase, as described in the section above [88,89,90,91]. HynSL from *A. macleodii* and the [NiFe] uptake hydrogenase from *T. roseopersicina* are highly identical [10,102]. Among the hydrogenase families, oxygen tolerance has been described among the [NiFe] hydrogenases, although not all [NiFe] hydrogenases are oxygen tolerant [21]. Though, some uptake [NiFe] hydrogenases are known to be oxygen-tolerant, they are generally directionally biased against hydrogen evolution [13,103]. An enzyme’s bias can be defined as the ratio of the maximal rates in either direction. What defines this bias and how it varies between essentially similar enzymes in different species remains to be elucidated. A case in point is the [NiFe] hydrogenases from *Allochromatium vinosum* (*A. vinosum*) and *Desulfovibrio fructosovorans* (*D. fructosovarans*)*.* The enzymes from both these organisms demonstrate properties typical of the [NiFe] class of hydrogenases, however they exhibit very different catalytic properties [104]. While the *D. fructosovarans* enzyme is an efficient catalyst for both hydrogen oxidation and formation, in *A. vinosum* the catalytic bias for hydrogen oxidation is at least ten times favored over the reductive capacity [105]. [NiFe] hydrogenases may thus serve as valuable models to understand the diverse mechanisms of tuning the reactivity of the hydrogen-activating site [17]. Site directed mutagenesis may help investigate the subtle relation between bias and structure within this family of enzymes.

The broad family of [NiFe] hydrogenase is comprised of two subunits, large and small. While the large subunit is home to the active site, the small subunit is believed to function as a molecular wire to guide the electrons to the active site. Generally, the frame work of the molecular wire comprises three linearly arranged, evenly spaced FeS clusters named after their proximity to the active site as proximal, medial, or distal. As a rule, the proximal and distal clusters are comprised of [4Fe-4S] while the medial cluster is a [3Fe-4S] cluster. The [4Fe-4S] clusters generally exhibit a lower midpoint potential than the [3Fe-4S] clusters. In *Desulfovibrio gigas* (*D. gigas*) the reported midpoint potential of the medial [3Fe-4S] cluster is −70 mV, while the proximal and distal midpoint clusters have midpoint potential of −290 and −340 mV, respectively [106]. The presence of a high potential [3Fe-4S] cluster right in the middle of the electron transfer chain came as a surprise when the crystal structure was first elucidated [106]. It was unclear how fast electron transfer could occur in the presence of a very “uphill” step. For a thermodynamically favorable transaction, the electrons must move from higher reduction potential to lower reduction potential. As such, the more negative the reduction potential, the greater is the species affinity to donate the electrons and be oxidized. Thus, the flow of electrons from the active site to the distal cluster via the medial cluster appeared thermodynamically unfavorable [8]. Hence the question whether the central cluster limits the rate of intramolecular electron transport from the [NiFe] center to the acceptor has been constantly debated. Dutton and coworkers have proposed that the only engineering principle of electron transfer chain that could explain such an electron transport would be the presence of a “small enough” inter-center distance that could transfer the electrons with a speed that made the reduction potentials of the clusters seem less influential [107,108]. Contrarily, if they were not serving a purpose, we may expect the redox potentials to vary with evolution. However, they appear to be conserved within each family of the enzyme [105].

Further questions arise of whether all the three [FeS]-clusters are actually involved in the process of transfer of electrons within the enzyme and whether the distal cluster acts as a natural point of entry/exit of electrons. Indeed it may be argued that the medial cluster may well possess the capacity to interact directly with the redox partner. However, this was ruled out in recent investigations with protein film voltammetry, where the enzyme was adsorbed onto the electrode and the electron transfer was direct [109]. The intrinsic bias of a catalyst is governed by the operational limits of the thermodynamics in a particular direction. Recent, literature is available where; enzyme bias in uptake hydrogenases was altered in experiments investigating the ligation of Fe-S clusters in the small subunit of [NiFe] hydrogenases [8,9,10,11].

Yonemoto *et al.* [10] aimed at introducing an orthogonal, oxygen tolerant, improved hydrogen production circuit into cyanobacteria. They introduced site directed mutations in the oxygen tolerant [NiFe] hydrogenase HynSL of *A. macleodii* HynSL which belongs to a group of membrane-associated hydrogenases classified as group 1 by Vignais *et al.* [2]. A 2% oxygen tolerance capacity makes this hydrogenase attractive in terms of its biotechnological application. Modification of ligands was based on two separate mutagenesis studies on the uptake hydrogenase from *D. fructosovorans* [8,9]. The authors introduced changes to both the medial and the distal cluster ligations. In the first such substitution the non-canonical proline 285 known to hold the [3Fe-4S] was replaced by cysteine. Proline 238 substitution to cysteine in HynA of *D. fructosovorans* induced the conversion of [3Fe-4S] cluster to [4Fe-4S] cluster [8]*.* The conversion changed the potential of the cluster from +65 to −250 mV. In addition, the conversion resulted in 30% reduction of hydrogen uptake activity and nearly a two-fold increase in hydrogen evolution. Simultaneous changes in the active site or potentials of the proximal and distal cluster were not observed. The study provided compelling evidence that the change in the cluster type could strongly influence the bias of the enzyme towards hydrogen production due to a change in the redox potential gradient. Coupled to this, another substitution was added where histidine 230, a protein ligand to the distal cluster, was substituted to cysteine. This was based on the importance of the distal cluster in influencing inter- and intra-molecular electron transfers. Substitution of histidine 184 to cysteine, in the distal cluster of the [NiFe] hydrogenase in *D. fructosovorans*, resulted in almost abolished uptake activity in the mutant while the evolution activity was only reduced by 50% [9]. Thus, Yonemoto *et al*. [10] introduced another substitution, replacing histidine 230 to cysteine. The variants of these two substitutions in *A. macleodii* hydrogenase were produced both individually and combined with the aim of improving the hydrogen production characteristics of the enzyme before introducing it into an oxygen evolving cyanobacteria [10]. Interestingly, changes introduced either to the medial cluster or the distal cluster decreased the hydrogen evolution and increased the uptake activity. However, the coupled influence of both the substitutions significantly enhanced hydrogen evolution while maintaining uptake activity constant. Concurrently, the thermal tolerance was slightly compromised while the oxygen tolerance of the hydrogenase was slightly enhanced. However, the effect of the substitutions on the composition of [FeS] clusters was not verified. Thus, at this stage it is difficult to comment whether indeed the changes were influenced by the change in the [FeS] cluster or whether more subtle changes in the electron transfer kinetics led to these changes. Interestingly the same effect was extrapolated in photosynthetic organisms emphasizing the importance of the development of dual systems for ease of screening. The study highlighted the importance of the histidine ligand in controlling the flow of the electrons into this system.

Yonemotto *et al.* [11] continued to work with the *A. macleodii* hydrogenase with an objective to obtain an enzyme that possessed still higher hydrogen evolving activity than their previous report. They carried out a broad survey of substitutions in HynSL of *A. macleodii,* where each of the 12 amino acid positions ligating the three Fe-S clusters in the small subunit was replaced by aspartic acid, histidine, asparagine, or glutamine, alternative coordinating ligands found in a broad survey of [NiFe] hydrogenases otherwise carrying conserved cysteine residue [11]. These substitutions were made in the background of the previous modified *A. macleodii* hydrogenase, GI strain. They found interesting trends in their preliminary screening of the mutants including, high level tolerance towards aspartic acid substitutions, while in certain hot spots including C78, C192 and C264 all substitutions resulted in abolished enzyme activity except for the aspartic acid substitutions which were generally well tolerated. Further proteomic analysis of the eight aspartic acid variants suggested that the activity differences in each of the reconstituted enzymes was due to differential enzyme processing as observed by the lack of small subunit *N*-terminal cleavage. The authors differentiated it as mature and immature forms of the enzyme and normalized the activity to the quantity of the mature small subunit observed in the gel to suggest improved hydrogen evolution over the parent strain [11]. This is a critical critique of this article. Also, no *in vitro* experiments were demonstrated. It would be interesting to look at the results within the regime of the native cellular metabolism*.* Also, these results were limited to *E. coli.* It would be interesting to see how mutagenesis affected the hydrogenase activity in cyanobacteria. In an initial study, the small subunit of the cyanobacterial HupSL uptake hydrogenase from the filamentous heterocystous strain *Nostoc punctiforme* was isolated through heterologous expression in *E. coli*. The protein was found to contain three FeS clusters, in accordance with previously isolated enzymes from other bacterial species. Although the FeS binding motifs show differences from earlier studied enzymes, their electron paramagnetic resonance signatures showed that the protein contains two [4Fe-4S] and one [3Fe-4S] cluster [110]. Future studies may address how different FeS cluster binding motifs affect the redox potentials of the three clusters.

In the context of cyanobacterial hydrogen production such studies may be effective in achieving better productivity using oxygen and thermo-tolerant enzymes. This may have added advantages over the oxygen labile [FeFe] hydrogenases.

## 3. Conclusions

Over the last decades, various aspects of cyanobacterial hydrogen evolving capacity have been studied. There are several contradicting reports present in the literature and several mechanisms still remain elusive. Attempts to introduce orthologous components to achieve higher enzymatic activity have shown only limited success so far. Internationally, consortiums have been funded to work on various aspects of this technology. There is a need to integrate the expertise of various groups at the fundamental and applied level to develop a novel cyanobacterial chassis that could be used as a self-sustained cellular factory to generate fuel.
